# Sexual function and wellbeing of women using modern contraceptive methods in Rwanda: a multicenter cross-sectional study

**DOI:** 10.3389/fgwh.2026.1776346

**Published:** 2026-02-11

**Authors:** Uwineza Mireille Aimee, Diomède Ntasumbumuyange, Polyphile Ntihinyurwa, Izere Salomon, Gerald Kaberuka, Aurore Nishimwe, Stephen Rulisa

**Affiliations:** 1 Department of Obstetrics and Gynecology, University of Rwanda College of Medicine and Health Sciences, Kigali, Rwanda; 2 Department of General Medicine and Surgery, University of Rwanda College of Medicine and Health Sciences, Kigali, Rwanda; 3 Centre for International Reproductive Health Training (CIRHT), Michigan University, Kigali, Rwanda; 4 Institute of Applied Health Research, University of Birmingham, Birmingham, United Kingdom

**Keywords:** contraception, family planning, FSDS-R, intrauterine device, reproductive health, Rwanda, sexual function

## Abstract

**Background:**

Family planning (FP) is essential for sustainable development, maternal health, and women's reproductive well-being. Despite its critical role, Rwanda continues to experience a highly unmet need for FP. Women's experiences and perceptions of contraceptive methods can significantly affect their adoption and use. This study aimed to assess the sexual function and well-being of women using modern FP methods in Rwanda.

**Methodology:**

A multicenter cross-sectional study involving 415 women aged ≥18 years who had used a modern FP method for at least ≥6 months was conducted across three urban and two rural FP clinics in Rwanda. Sexual function and well-being were evaluated via the Female Sexual Distress Scale-Revised (FSDS-R), with scores <11 indicating good sexual function. Utilizing R programming version 4.0.2, Logistic regression was used to examine associations between demographic/clinical factors and sexual distress.

**Results:**

Overall, 79.5% of women had good sexual function (FSDS-R < 11). Copper intrauterine device (IUD) users (96%) and those with permanent sterilization (84%) reported the highest sexual satisfaction. In multivariable analysis, underweight women (BMI <18.5) had 3.08-fold higher odds of sexual distress than normal-weight women (OR = 3.08; 95% CI 1.10–8.69). Conversely, IUD users had 86% lower odds of distress than implant users (OR = 0.14; 95% CI 0.03–0.42). Other factors, such as education level, were not significantly associated after adjustment.

**Conclusion:**

The majority of Rwandan FP users in this study reported satisfactory sexual function. Contraceptive method and BMI were key predictors. These findings underscore the importance of integrating sexual health counseling into FP programs to reassure women about contraceptive side effects and support informed method choices that optimize reproductive well-being.

## Introduction

Sexual health is a fundamental aspect of overall well-being (1, 2), encompassing the physical, emotional, and social dimensions. This includes sexual function, satisfaction, and reproductive health. Family planning (FP) methods play a crucial role in sexual health by enabling individuals and couples to make informed decisions regarding reproduction (3) with discontinuation potentially leading to higher fertility rates (4, 5), although the impact can vary among different methods (6, 7).

Sexual health issues affect a substantial proportion of women, with estimates ranging from 30%-50%, leading to notable physical and emotional challenges (8, 9). The World Health Organization (WHO) recognizes women's sexual health as a fundamental human right (2). Contraceptives aim to prevent pregnancy while enhancing overall health and sexual experiences (10). They can influence sexual function and satisfaction in several ways, and decisions regarding the use or discontinuation of contraceptives are often influenced by their effects on sexual pleasure and overall satisfaction during sexual activity (1, 11, 12).

Among the 1.9 billion women in the reproductive age group (15–49 years) worldwide in 2021, 1.1 billion needed family planning; of these, 874 million used modern contraceptive methods, and 164 million had an unmet need for contraception (7, 13). In developing countries and certain religious communities, modern FP faces barriers such as limited knowledge, spousal opposition, misconceptions, restricted access, and concerns about side effects, particularly sexual disturbances, etc., following the use of contraceptives (14–17) Despite their benefits, women's concerns about side effects often outweigh their perceived advantages (18), exacerbated by misconceptions and rumors within communities (4, 5). In Rwanda, for example, 34% of FP users discontinued their methods owing to side effects (19). Similar trends have been reported in other regions, with side effects reported by 54.6% of women in Egypt (20) and varying rates in Tanzania reporting side effects of pills and injections (21). These side effects can significantly affect the sexual health and overall well-being of modern contraceptive users (1, 10, 20, 22, 23). FP side effects occur during the first two to three months, after which the body is familiar with the method, and it is an ideal time to assess sexual dysfunction associated with modern family planning methods (24).

A review by Casey et al. indicated that while many studies have shown that, compared with nonuse, contraception generally improves sexual function, the results are mixed (25). For instance, combined oral contraceptives (OCPs) have been associated with reduced libido but stable sexual satisfaction over time. Conversely, some studies have reported increased sexual desire with the use of OCPs or copper IUDs (25). However, other studies have highlighted the significant changes in sexual life and quality of life associated with hormonal contraceptives (1, 2, 10).

The impact of contraceptive methods on sexual health remains inconsistent (20, 21, 26), with some research suggesting benefits such as reduced dysmenorrhea and anemia (23, 27) and others documenting declines in sexual desire and satisfaction (10, 23). These varying effects influence women's choices of contraceptive methods and their continued use (1, 11, 12).

Rwanda, one of Africa's most densely populated and rapidly growing countries in the region (28), views family planning as a crucial component of its economic development strategy (29). From 2005 to 2020, the use of modern contraceptives in Rwanda increased significantly from 17% to 58% (19, 22, 30). However, the 2014–2015 Rwanda Demographic and Health Survey (RDHS) indicated only a modest increase of approximately 3% in modern contraceptive use over the previous five years (22). The RDHS 2019–2020 reported a 12-month discontinuation rate of 30%, with health concerns being the primary reason for discontinuation (31).

To date, no comprehensive study has assessed the sexual well-being of modern contraceptive users in Rwanda. This is the first study that explored how modern FP methods influence sexual function, satisfaction, and overall sexual well-being among Rwandan women. Understanding these dynamics will enable healthcare providers to better support women in making informed family planning choices and addressing related concerns.

## Methodology

### Study design

This study employed a multicenter cross-sectional design to assess the sexual well-being of women using modern contraceptive methods in Rwanda. Participants were recruited consecutively.

### Setting

The research was conducted across multiple sites, including family planning clinics in both rural and urban settings. The University Teaching Hospital of Kigali (CHUK) is located in the capital city of Kigali, within the Nyarugenge district (32). It is the largest referral hospital in Rwanda. It serves as a primary facility for high-risk pregnancies in various district hospitals in Kigali, offering long-acting contraceptive options and permanent sterilization for patients. Since its establishment in May 2019, the CHUK's FP clinic has consistently served approximately 70 women per month.

Health centers (HC) in Rwanda function as primary healthcare facilities, delivering most of the FP services within local communities due to their accessibility (33). They serve a large number of the population, ensuring wide coverage of FP services. For this study, we selected four health centers, two in urban areas (Kacyiru and Muhima) and two in rural areas (Nyamata and Remera Rukoma) to explore how differing lifestyles might influence sexual health perceptions among women.

The Kacyiru HC, which initiated FP services in 2010, serves an average of 250 women per month. The Muhima HC, which has been active in FP services since 2000, attends to approximately 300 women monthly. The Nyamata HC, which is in a rural area and has offered FP services since 2005, also serves 250 women per month. The Rukoma HC, which began FP services in 2000, has the highest average, serving approximately 400 women monthly. The selection of these health centers allowed us to compare the experiences and perceptions of women in urban vs. rural settings, shedding light on the potential impact of lifestyle differences on sexual health.

### Eligibility criteria

We enrolled a total of 415 women in the study. Women aged 18 years or older who had been using one of the following modern contraceptive or family planning methods for at least six months: hormonal contraceptives (oral pills, injectables, implants), IUDs, permanent methods (tubal ligation), etc., were included in the study. All eligible women attending the family planning clinics during the data collection period were invited to participate. At each facility, trained research staff approached women on clinic days and enrolled those who met the inclusion criteria and provided consent. We did not use a strict random sampling scheme, but rather aimed to include all consenting eligible women during the study timeframe. The exclusion criteria included women under the aforementioned years of age and individuals with chronic illnesses or who used traditional family planning methods.

### Sampling method

The sample size for this study was calculated using the Taro Yamane formula (Yamane, 1973), with a 95% confidence level and a margin of error of 0.05. The formula used is as follows:

$$n=\frac{N}{1+N*{\left(e\right)}^{2}}$$

Where **N** represents the population size, **n** denotes the sample size, and **e** is the margin of error.

For this study, the population size (**N**) was 17,000, corresponding to the number of women who visited FP clinics from June 2021 to June 2022. Based on this calculation, the required sample size was determined to be 391, and we chose to recruit 415 women.

Recruitment was conducted by consulting the FP clinic registers to obtain contact information. Eligible women were contacted via telephone, informed about the study objectives, and invited to participate. Those who agreed to participate were scheduled for appointments at their respective health facilities. On the scheduled date, a research assistant conducted a brief introductory session to explain the aims, expected outcomes, and procedures of the study. The participants were allowed to ask questions, which were addressed by a research assistant. Written informed consent was obtained from each participant before the commencement of data collection.

### Data collection tools

**Figure 1:**
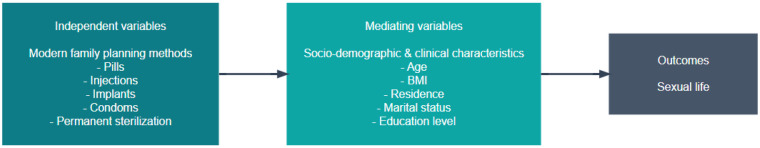
Concept framework (model by JIE HU).

Two primary instruments were used for data collection. A predesigned questionnaire gathered demographic and clinical information, including age, BMI, marital status, religion, education, parity, and the type of contraceptive method used (Figure 1), as detailed in Supplementary File S1. The second tool was the Female Sexual Distress Scale-Revised (FSDS-R), a validated 13-item questionnaire that measures sexually related personal distress in women (34, 35). The FSDS-R was developed and validated by DeRogatis et al. (2008) for use in women with sexual concerns (35).

Participants rated items on a 0–4 scale, with higher scores indicating greater distress. The FSDS-R total score ranges from 0 to 52, with a score of 11 or higher indicating potential sexual dysfunction. The FSDS-R was professionally translated into Kinyarwanda for cultural relevance. Prior cross-cultural validation studies have demonstrated good to excellent internal consistency, with Cronbach's *α* ranging from 0.83 to 0.95 across diverse language versions (Turkish, Brazilian Portuguese, Arabic) (36–39).

### Data analysis

The data analysis in this study integrated descriptive statistics, inferential analyses, and regression modeling to comprehensively examine the relationships among sociodemographic factors, FP methods, and sexual function outcomes. Initially, descriptive statistics, which are presented as the means, standard deviations, and proportions for variables such as age, BMI, marital status, education level, and FP method, were used to summarize the participants’ characteristics. To identify significant associations between categorical variables, chi-square tests were conducted, revealing links between educational attainment, BMI categories, FP choices, and sexual function scores. Additionally, inferential statistics were used to assess differences in sexual function across various demographic groups. To further elucidate the predictors of sexual distress, logistic regression analysis was performed, allowing for the simultaneous evaluation of the impact of multiple independent variables while controlling for potential confounders. Variables significant at *p* < 0.20 in univariable analysis were entered into multivariable logistic regression models. Missing data accounted for less than 5% and were handled using complete-case analysis. All the statistical analyses were conducted using the R programming version 4.0.2. Statistical significance was set at a *p*-value of <0.05.

### Ethical considerations

This study was approved by the Institutional Review Board (IRB) of the College of Medicine and Health Sciences, University of Rwanda (No 189/CMHS IRB/2022), and by the hospital's ethical committee. All participants signed informed consent, affirming their understanding of the study's objectives, their right to confidentiality, and their freedom to withdraw from the study at any stage without consequences.

## Results

### Demographic characteristics

This study included 415 women who met the inclusion criteria. The detailed sociodemographic characteristics are presented in Table 1.

**Table 1:** Demographic characteristics of the study participants.

Variable	Categories	*N* (%)	Mean (SD)
Respondent age group	18–24	59 (14.2)	
	25–31	167 (40.2)	
	32–38	137 (33)	
	39–49	52 (12.5)	
Type of residence	Rural	187 (45.1)	
	Urban	228 (54.9)	
Marital status	Divorced	8 (1.9)	
	Married	371 (89.4)	
	Single	33 (8)	
	Widowed	3 (0.7)	
Religion	Christian	398 (95.9)	
	Muslim	14 (3.4)	
	Traditional	3 (0.7)	
Insurance status	Has health insurance	408 (98.3)	
	No life insurance	7 (1.7)	
Education level	High school	215 (51.8)	
	None	11 (2.7)	
	Primary	131 (31.6)	
	University	58 (14)	
Number of Children	"1–2"	240 (57.8)	
	"3–4"	136 (32.8)	
	more than 4	36 (8.7)	
	None	3 (0.7)	
BMI (kg/m^2^)	Underweight (<18.5)	19 (4.6)	
	Normal (18.5–24.9)	186 (44.8)	
	Overweight (25–29.9)	145 (34.9)	
	Obese (above 30)	65 (15.7)	
	Age of patients		31 (6.71)
	Weight of the patient		66.8 (12.6)
	Height of the patients		161.8 (7.91)
	BMI of the patients		25.12 (4.56)

About 54.9% of women were from urban areas and aged between 25 and 38 years (73.2%), with a mean age of 31 years (*SD: 6.71*). Most participants were married (89.4%) and completed high school (65.8%). A significant proportion of respondents had life insurance (98.3%). Most participants had one to four children (90.6%). The average BMI of the participants was 25.12 kg/m^2^ (*SD: 4.56*), with less than half of the study population (44.8%) falling within the normal BMI range (18.5–24.9 kg/m^2^). The mean weight of the women was 66.8 kg (*SD: 12.6*).

### Utilization of family planning services

**Figure 2:**
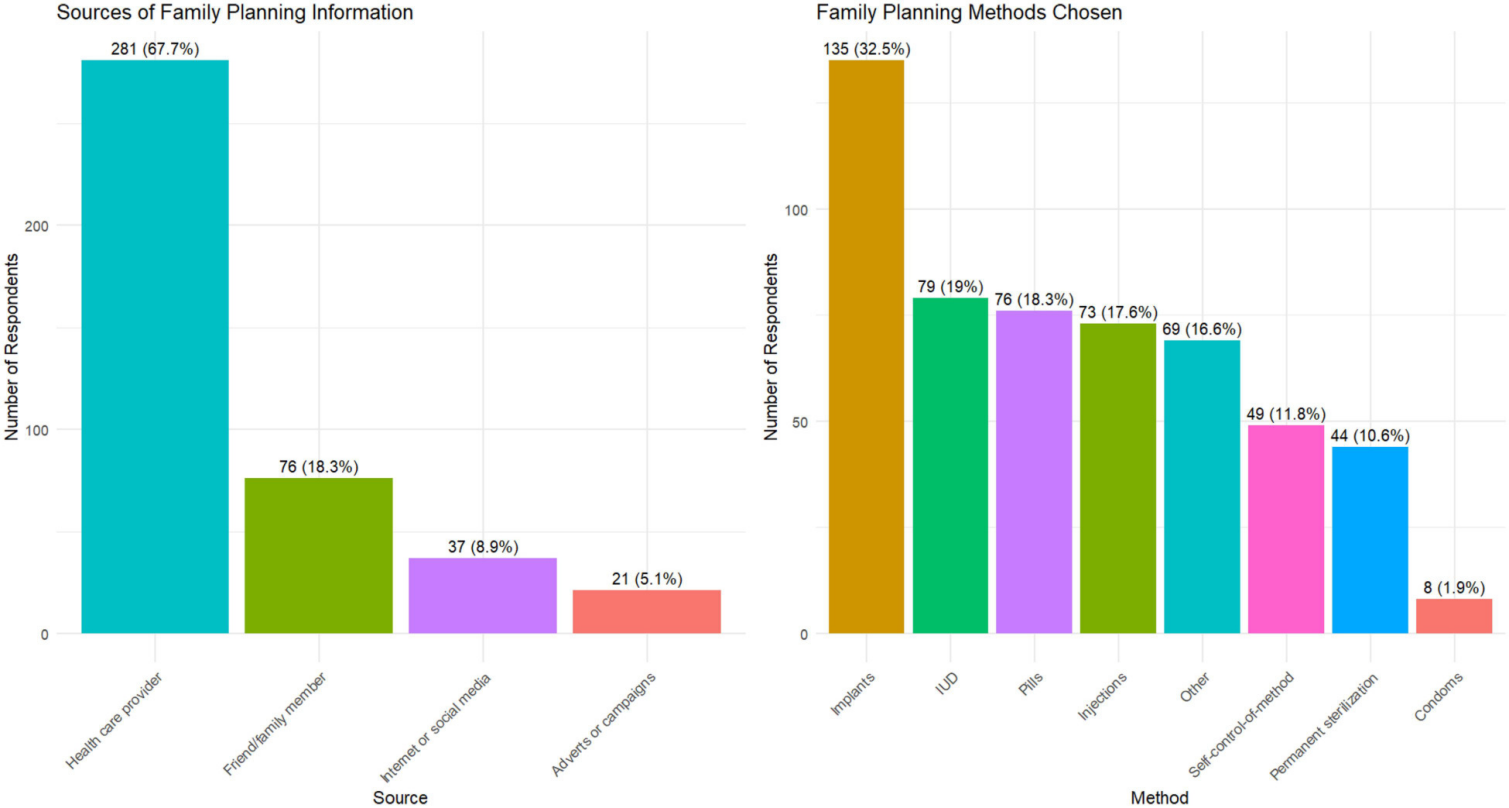
Utilization of family planning services and source of information.

The majority of family planning information was obtained from healthcare providers (67.7%), with family and friends serving as secondary sources (18.3%) (Figure 2). In terms of contraceptive methods, implants emerged as the most widely used option, chosen by 32.5% of women. This was followed by IUDs at 19%, oral contraceptive pills at 18%, injections at 17.6%, permanent sterilization at 10.6%, and condoms at 1.9%.

### Data related to sexual dysfunction and its associated factors

#### Sexual function/well-being data

Among women using modern family planning methods, 79.5% reported good sexual satisfaction, with FSDS-R scores at or below 11 points. Sexual function scores were notably better in the 32–38 years (mean score: 4.93, *SD: 10.51*) and 39–49 years (mean score: 4.65, *SD: 10.16*) age groups. In contrast, the younger age group (18–24 years) presented poorer sexual function (mean score, 7.98; *SD: 13.15*). Compared with their rural counterparts, urban women demonstrated better sexual function, with scores of 5.21 (*SD: 10.99*) vs. 6.53 (*SD: 11.01*), respectively (Table 2). Sexual functioning also varies significantly according to marital status. Widowed and single women had better sexual function scores than married and divorced women, with mean scores of 2 (*SD: 3.46*) and 4.12 (*SD: 9.83*), respectively. Married women had a mean score of 5.82 (*SD: 10.98*), whereas divorced women had notably poorer sexual function, with a mean score of 13.25 (*SD: 16.46*), indicating a real sexual dysfunction and highlighting the potential impact of social and cultural factors on sexual function. This is particularly relevant in societies where marital status is closely tied to religious beliefs and where sexual activity outside marriage is discouraged or stigmatized. Such factors may contribute to poorer sexual function in divorced women. Healthcare providers should offer tailored sexual health counseling for divorced and single women, who may face heightened social stigma regarding sexual activity and contraceptive use, potentially contributing to higher sexual distress. Notably, the proportion of single and widowed participants was relatively small.

**Table 2:** Summary of the sexual function scores of the respondents by demographic characteristics.

Characteristics	Categories	Range	Mean (SD)
Age group	18–24	0–48	7.98 (13.15)
	25–31	0–52	6.11 (10.81)
	32–38	0–48	4.93 (10.51)
	39–49	0–52	4.65 (10.16)
Type of residence	Rural	0–48	6.53 (11.01)
	Urban	0–52	5.21 (10.99)
Marital status	Divorced	0–45	13.25 (16.46)
	Married	0–52	5.82 (10.98)
	Single	0–41	4.12 (9.83)
	Widowed	0–6	2.00 (3.46)
Level of education	High school	0–51	5.34 (10.47)
	None	0–48	9.55 (17.37)
	Primary	0–52	8.06 (12.38)
	University	0–29	1.72 (5.85)
BMI classification	Normal	0–51	5.96 (11.16)
	Obese	0–52	5.89 (12.65)
	Overweight	0–52	4.51 (9.06)
	Underweight	0–45	13.89 (14.14)
Number of children	"1–2"	0–52	6.77 (11.81)
	"3–4"	0–48	4.25 (9.68)
	more than 4	0–42	5.44 (10)
	None	0–11	3.67 (6.35)
Type of method	Condoms	0–3	0.38 (1.06)
	Implants	0–52	8.49 (13.30)
	Injections	0–51	8.63 (12.35)
	IUD	0–33	1.35 (4.68)
	Permanent sterilization	0–42	4.20 (10.03)
	Pills	0–48	4.45 (8.82)

Educational attainment was positively associated with sexual function; women with university-level education had the best scores (mean score: 1.72, *SD: 5.85*), whereas scores declined with lower educational levels. The group with no formal education had the poorest sexual function (mean score: 9.55, *SD: 17.37*).

BMI categories revealed that overweight, obese, and normal-weight women had comparable favorable sexual function scores. In contrast, underweight women had significantly poorer scores (mean score: 13.89, *SD: 14.13*) than did their normal weight (mean score: 5.96, *SD: 11.16*), overweight (mean score: 4.51, *SD: 9.06*), and obese counterparts (mean score: 5.89, *SD: 12.65*) (Table 2).

Women with 3–4 children reported better sexual function scores (mean score: 4.25, *SD: 9.16*) than those in the other groups. Most participants had 1–2 children (57.8%), followed by 3–4 children (32.8%). The groups with more than four children (8.7%) and no children (0.7%) were too small for meaningful comparison with larger groups (Table 2). Concerning FP methods, women whose husbands used condoms had the highest sexual function scores (mean score, 0.38; *SD: 1.06*). This was followed by IUDs (mean score, 1.35; *SD: 4.68*) and permanent sterilization (mean score, 4.2; *SD: 10.03*). Women using injections had the lowest sexual function scores (mean score: 8.63, *SD: 12.35*) (Table 2).

### Sexual function and its associated factors

A chi-square test was performed to assess the relationships between FSDS-R scores and various demographic characteristics. The findings are summarized in Table 3.

**Table 3:** Correlations between female sexual distress and the characteristics of the respondents.

Characteristics	Categories	*N* good	*N* Poor	% of good sexual function	*X*^2^ (DF)	*P* value
Age group	18–24	41	18	69.4		0.213
	25–31	134	33	80.2	4.484 (3)	
	32–38	113	24	82.5		
	39–49	42	10	80.7		
Type of residence	Rural	144	43	77	1.0536 (1)	0.304
	Urban	186	42	82		
Marital status	Divorced	5	3	63		0.298
	Married	293	78	79	3.6786 (3)	
	Single	29	4	88		
	Widowed	3	0	100		
Education level	None	8	3	73		**0** **.** **003**
	High school	176	39	82	14.549	
	Primary	92	39	70		
	University	54	4	93		
BMI classification	Normal	146	40	78		**0**.**015**
	Obese	52	13	80	10.462	
	Overweight	122	23	84		
	Underweight	10	9	53		
Number of children	1–2	185	55	77		0.644
	3–4	115	21	85	2.5025 (4)	
	more than 4	28	8	78		
	None	2	1	67		
Type of method	Implants	98	37	73		**<0**.**001**
	Condom	8			27.188	
	Injections	49	24	67		
	IUD	76	3	96		
	Permanent sterilization	37	7	84		
	Pills	62	14	82		

Bold *P* values are significant.

The chi-square test revealed a significant association between educational attainment and sexual function. Women with university-level education demonstrated higher sexual function scores than those with only high school education or no formal schooling (*p* = 0.003). BMI was positively correlated with the sexual function score (*p* = 0.015). Underweight women (BMI < 18.5) had the poorest sexual function scores, whereas overweight women had the highest scores. Notably, 53% of underweight women reported satisfactory sexual function, whereas 84% of overweight women reported satisfactory sexual function, regardless of the family planning method used.

**Figure 3:**
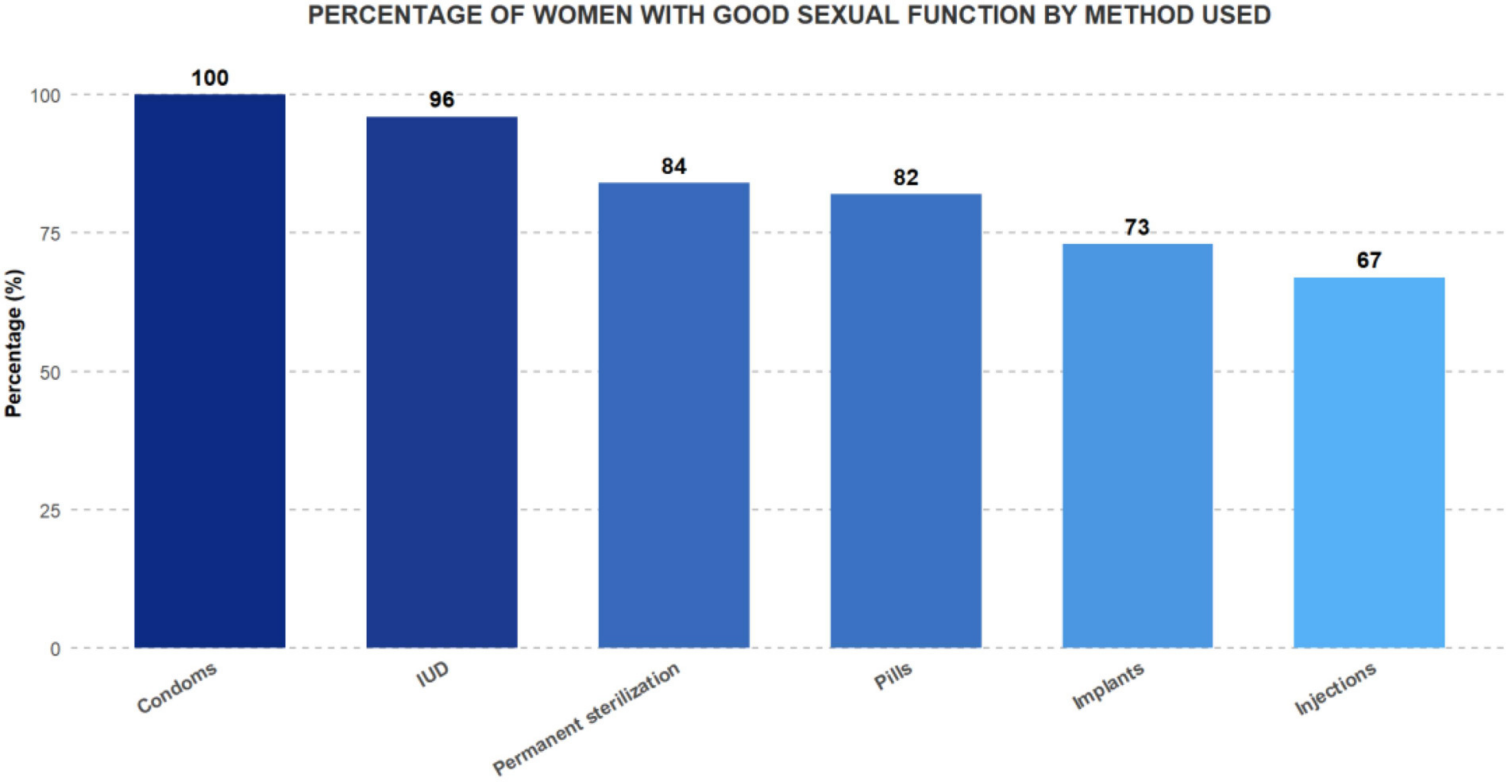
Percentage of women with good sexual function by type of FP method used.

The type of contraceptive method also significantly affected sexual function scores (*p* = 0.00049). Users of condoms and IUDs reported better sexual function, with 100% and 96% of these women scoring above the FSDS-R cutoff of 11 points. Permanent sterilization followed, with 84% exceeding the cutoff. Conversely, the injection users had poorer sexual function scores (Figure 3). Although condom users demonstrated 100% good sexual function, this finding should be interpreted with extreme caution due to the very small sample size (*n* = 8).

Further analysis via logistic regression was used to assess the associations between various sociodemographic variables and female sexual distress levels. The results indicated that, in addition to underweight status, which was significantly associated with greater female sexual distress, the other factors did not have statistically significant associations. Specifically, underweight women were 3.08 times more likely to experience female sexual distress than women with a normal weight [CI = (1.10, 8.69)]. Additionally, women using IUDs were 86% less likely to experience sexual distress than those using implants [CI = (0.03, 0.42)] (Table 4).

**Table 4:** Predictive factors of good sexual health.

Characteristics	Categories	*P* value	OR	95%CI
Education level	None (refcat)		1	
	High school	0.52	0.63	[0.16–3.10]
	Primary	0.22	0.56	[0.21–1.36]
	University	0.22	0.34	[0.06–2.10]
BMI classification	Normal (refcat)		1	
	Obese	0.50	0.43	[0.31–1.36]
	Overweight	0.36	0.76	[0.41–1.37]
	Underweight	0.03	3.08	[1.10–8.69]
Type of method	Implants (refcat)		1	
	Injections	0.29	1.42	[0.74–2.70]
	IUD	0.00	0.14	[0.03–0.42]
	Permanent sterilization	0.22	0.56	[0.21–1.36]
	Pills	0.50	0.78	[0.37–1.58]

## Discussion

### Key findings

This study investigated the sexual well-being of women using modern family planning methods in Rwanda, focusing on how these methods affect sexual satisfaction and associated sociodemographic characteristics. Our findings suggest that women who use modern contraceptives generally report satisfactory sexual experiences.

Higher educational levels were positively associated with improved sexual function scores.

This aligns with the findings of Abdallah et al. (40) who noted that Egyptian women with higher educational levels experienced better sexual function. They reported that women with MSc and MD had better sexual fulfillment and less dyspareunia than did those with lower education levels, which could be due to enhanced knowledge and coping strategies related to sexual and reproductive health (40). Conversely, Gabalci et al. (41) used the Arizona Sexual Experience Scale (ASEX) to score women via the family planning method and reported that lower education levels were associated with higher sexual function scores among Turkish women. Higher scores were observed for primary school education or lower education, and the statistical comparison with the scores of the higher education groups was significant (*p-value < 0.05*) (41). This discrepancy might be due to different cultural contexts and the varying impacts of education on sexual health perceptions in different populations. Although education level was associated with sexual function in bivariate analysis, it acted as a confounder in multivariable models, particularly influencing the relationship between contraceptive method choice and sexual distress.

This study (41) Also demonstrated that being over 31 years old was correlated with higher sexual function scores, similar to the higher sexual function scores reported in the 32–38 years old (41) Age group, possibly due to the fulfillment of women in the age group above 30 years, as they are employed and settled in families.

Our study revealed that 96% of women using IUDs achieved satisfactory sexual function scores, which is consistent with the findings of Gabalci et al. (41, 42). Similar positive outcomes for IUD users were also reported by Akintomide and Brima, who found that IUDs were associated with increased sexual satisfaction among women in the UK (43). In contrast, women using implants reported lower sexual function scores, which is consistent with results from Moreira et al., who revealed that nonhormonal LARCs significantly preserved sexual function compared with etonogestrel implants, with a strong significant difference (*p* < 0.001) (42). This contrasts with the findings of Guida et al. (44), who reported that OCPs were associated with better sexual function in approximately 44% of Italian women than in 36% of IUD users among Italian women (44).

It is also important to consider biological differences between contraceptive methods. Hormonal methods, especially progestin-only injectables and implants, have been linked to reduced sexual desire in some studies (45), possibly due to their endocrine effects (e.g., lowering free testosterone). In contrast, non-hormonal methods such as the copper IUD are not associated with altered libido (46). For example, in a large US cohort study, women using progestin injections or implants reported higher odds of decreased interest in sex compared to copper IUD users (45), while no effect was seen for combined oral contraceptives or hormonal IUDs. Our finding that copper IUD users reported lower sexual distress than some hormonal users is consistent with this. These physiological differences may partly explain variations in sexual wellbeing across contraceptive types.

Our study also revealed that 82% of women using OCPs reported good sexual function, which is consistent with the findings of Casey et al. (21) and Guida et al. (25, 44), who reported improvements in sexual function among women using OCPs and the Levonorgestrel vaginal ring after 3 months of uptake (25, 44).

Additionally, Casey et al. compared 150 Iranian women who had tubal ligations to 150 who used condoms and reported that women who underwent tubal ligation had poorer sexual function scores than did those who used condoms, with poorer FSFI ratings across all domains; surprisingly, compared with women who did not regret their tubal ligation, FSD rates were greater in the latter group (25). This finding is similar to our findings, in which women with permanent sterilization had lower sexual function scores, though we did not distinguish between those who regretted or not regret the procedure. On the other hand, Di Carlo et al. (24) Observed significant improvements in sexual function among women using the etonogestrel-releasing implant (Nexplanon) (*p* < 0.01) after three months, which is corroborated by our study showing that 73% of implant users achieved satisfactory sexual function (24).

Overall, our findings are consistent with the global literature on the sexual function of women using modern FP methods. Studies conducted in diverse settings, such as those by Moreira et al. (42) and Casey et al. (25), have demonstrated that the choice of contraceptive method plays a significant role in shaping sexual function and satisfaction. Globally, the use of non-hormonal contraceptives, such as IUDs, has been associated with higher sexual satisfaction and fewer adverse sexual outcomes compared to hormonal methods, as observed in the studies of Akintomide and Brima (43) and Guida et al. (44). While hormonal contraceptives like OCPs and implants show mixed results depending on the population and specific method, the overall trend suggests that family planning, when tailored to individual needs, can positively influence sexual well-being. These findings reinforce the importance of considering cultural, educational, and individual factors when advising women on family planning choices, ultimately supporting better sexual health outcomes.

### Strengths and limitations of the study

This study utilized the FSDS-R, a widely recognized and validated tool for assessing the sexual function of women. Administering these questionnaires in Kinyarwanda, the local language, facilitated more effective communication with the participants and ensured accurate responses. The use of these established instruments allows comparability with other research findings. Efforts were made to minimize missing data and enhance the reliability of the findings.

However, this study has limitations; comparative analysis with a control group of non-contraceptive users would provide a more comprehensive understanding of the impact of contraceptive methods on sexual function. Moreover, a qualitative approach can offer deeper insights into the subjective experiences and perceptions of contraceptive users. The findings of this study are specific to the populations at the selected study sites; a larger nationwide sample would increase the generalizability of the results. Additionally, the study's cross-sectional design did not allow for the evaluation of changes over time, which could be addressed through longitudinal follow-up in future research, furthermore, Because of the cross-sectional design, baseline sexual function prior to contraceptive initiation could not be assessed. Longitudinal studies with pre-contraception measurements would better isolate the effects of specific FP methods from pre-existing sexual health conditions.

## Conclusion

This study provides valuable insights into the sexual health outcomes associated with modern family planning methods in Rwanda. The results indicate that women using these methods generally experience satisfactory sexual function, with significant influences from the type of contraceptive, educational level, and BMI. Compared with other contraceptive methods, IUDs are associated with better sexual function outcomes.

To increase the effectiveness of FP programs, emphasizing the benefits of modern contraceptive methods through targeted campaigns and counseling is essential. Such initiatives should also address and dispel prevalent myths and misconceptions about contraception, as the data suggests that sexual function is not adversely affected, as is commonly believed.

Moreover, advancing educational attainment among women should remain a priority, as it has a positive impact on sexual health outcomes. Equally important is the promotion of healthy lifestyle choices to maintain a normal BMI, which contributes to better sexual function. Future research should focus on longitudinal studies to further investigate the long-term effects of modern family planning methods and the evolving dynamics of contraceptive practices among women in Rwanda.

## Data Availability

The original contributions presented in the study are included in the article/Supplementary Material, further inquiries can be directed to the corresponding author.
