# Supplementary material for: Sexual function and wellbeing of women using modern contraceptive methods in Rwanda: a multicenter cross-sectional study
Source: Front Glob Womens Health. 2026 Feb 11;7:1776346. doi: 10.3389/fgwh.2026.1776346 (PMC12932436; doi:10.3389/fgwh.2026.1776346)
Supplement: Supplementary file 1 [file Datasheet1.pdf]

SEXUAL LIFE IN WOMEN ON MODERN FP METHODS IN RWANDA

Data collection tool (Demographic and clinical data)

Please read and fill the information cautiously, consider prescribed units of measures and select one appropriate answer if provided. Where the option "other" is the best, please write down the actual response

**Variable Code**<br>(Short name for the variable in the dataset)	**Variable Label**<br>(Longer description of what is included in the variable)	**Value Label**<br>(For categorical variables, what are the different levels of your variable, and what number will you assign to that level)
ID	Patient ID,<br>**Numero imuranga**	
AgeG	Age group<br>**Imyaka**	1. 18 – 24<br>2. 25 – 31<br>3. 32 – 38<br>4. 39 – 49
Weight	Weight of patient<br>**Ibiro**	In Kg
Height	Height of patient<br>**Uburebure**	In Cm
BMI	Body mass index	In m^2/kg
Residency /Address	Place where the patient lives: District/sector/cellular/ village<br>**Aho atuye**	1. Village / **Icyaro**<br>2. City / **Umujyi**
MaritalS	Marital status<br>**Icyiciro cy'irangamimerere**	1. Single / **sindashaka**<br>2. Married / **narashatse**<br>3. Divorced / **natandukanye nuwo twashakanye**<br>4. Widowed / **narapfakaye**
Religion	Religion of the woman<br>**Idini**	1. Christian/ **umukristu**<br>**2.** Muslim / **umusilamu**<br>3. Traditionnal /**gakondo**
Insurance	Insurance status<br>**Ubwishingizi mu kwivuza**	**1.** Mutuelle de Sante/ **Ubwisungane mu kwivuza**<br>2. Private/ **nta bwishingizi**
Education	Level of education<br>**Amashuri yize**	1. University level/ **Kaminuza**<br>2. High school/ **Amashuri makuru**<br>**3.** Primary school/ **Amashuri mato**<br>4. None/ **Sinize**
Parity	How many children do you have?	1. No child/ **Nta mwana mfite**<br>2. 1 – 2

	**Abana Afite**	3. 3 – 4<br>4. More than 4 / **hejuru ya bane**
Info	How did you get the information on family planning services?<br>**Ni mu buhe buryo wamenye ibijyanye no kuboneza urubyaro?**	1. Adverts or campaigns/ **Imbwirwaruhame cyangwa kwamamaza**<br>2. Health care provider / **Abaganga**<br>3. Internet or social media/ **Itangazamakuru**<br>4. Friend or family member/ **Inshuti cyangwa umuvandimwe**
RSeeking	Reasons for seeking FP services<br>**Ni ukubera iki waje kuboneza urubyaro?**	1. Need for spacing births/ **Gutandukanya urubyaro**<br>2. Have sex without children/ **Gukora imibonano mpuzabitsina simbyare**<br>3. Economic burden of children/ **Ibibazo by'imibereho y'abana (kubagaburira, kwiga, kuvurwa,…)**<br>4. Pregnancy related complications / **Ibibazo bijyanye no gutwita**<br>5. To prevent pregnancy and STIs/ **Kwirinda gusama hamwe n'indwara zandurira mu mibonano mpuzabitsina**<br>**6.** Husband pressure/ **Gutitirizwa n'umugabo**<br>7. Social pressure/ **Gutitirizwa n'abo tubana muri societe**<br>8. Restrictions from government services/ **Gukumirwa muri gahunda zimwe na zimwe za leta**
TypeFP	Which types of FP method did you choose?<br>**Ni ubuhe buryo wahisemo gukoresha?**	1. Pills/**Ibinini**<br>2. Injections/**Inshinge**<br>**3.** Implants/ **udupira two mu kaboko**<br>**4.** IUD/ **Agapira ko mu mura**<br>5. Condoms /**Agakingirizo**<br>6. Permanent sterilization/ **Gufunga urubyaro bya burundu**<br>7. Other…./ **Izindi mpamvu**
RChoice	On which reasons did you	**1.** No procedure required/ **Nta**

	choose that method<br>**Kuki aribwo buryo wahisemo ?**	**kubagwa bisaba**<br>**2.** Non invasive/ **ntibyangiza**<br>**3.** Hormonal /**Harimo imisemburo**<br>4. Non hormonal / **Nta misemburo irimo**<br>**5.** Less follow up required/ **Ntibisaba gukurikiranwa kenshi**<br>6. Self-control of the method/ **Uburyo ushobora kugenzura igihe cyose**<br>7. STI prevention/ **kwirinda indwara zandurira mu mibonano mpuzabitsina**<br>8. Other…../ **Izindi mpamvu**
